# Intercostal Nerve Transfers to Native Triceps or Free Muscle Flaps for Elbow Extension in Brachial Plexus Injuries

**DOI:** 10.1055/s-0043-1778063

**Published:** 2024-01-22

**Authors:** Scott Ferris, Simon Maciburko

**Affiliations:** 1 Plastic, Hand and Faciomaxillary Surgery Unit, The Alfred Hospital, Victoria, Australia; 2 Victorian Plastic Surgery Unit, St Vincent's Private Hospital Melbourne, Victoria, Australia

**Keywords:** intercostal nerve, brachial plexus, nerve transfer, free-functioning muscle transfer

## Abstract

Intercostal nerve donors for traumatic brachial plexus injury reconstruction have been used to neurotize native muscles or free-functioning muscle transfers, with inconsistent outcomes reported. The aim was to record a substantial series, evaluate functional outcomes, and identify prognostic factors. We present a single-surgeon case series of 21 consecutive patients who underwent 21 transfer procedures to either native muscles or free-functioning muscles to reconstruct elbow extension over a 9-year period. Outcome parameters included target muscle power grade and timing of recovery. A Medical Research Council power grade ≥ M4 was achieved in 17 reconstructions. The free-functioning muscle group had significantly higher success rate and reached their best power grade 14 months earlier. Free-functioning muscle reconstruction with intercostal nerve transfer is a more complex procedure but has quicker functional recovery and greater reliability in achieving grade M4.

## Introduction

Traumatic brachial plexus injuries (BPIs) have a spectrum of debilitating morbidity that create complex reconstructive challenges for upper limb surgeons. In current practice, combinations of nerve grafting, nerve transfers, and/or free-functioning muscle transfer (FFMT) are often required to regain a useful upper limb. 1 2 3 The limited availability of proximal donor nerves requires surgeons to identify and utilize other donors outside the ipsilateral brachial plexus and zone of injury, such as the intercostal nerves (ICNs), spinal accessory nerve, phrenic nerve, and/or the contralateral C7 nerve root. 4 5 6

The ICN as a donor for BPI reconstruction was first described by Seddon in 1963 and has since become a widely used donor to neurotize native muscles or FFMTs, with or without nerve grafting. 7 8 9 10 11 12 Some authors avoid using ICNs completely if a nerve graft will be required. 13 14 15 Outcomes reported after ICN transfers are inconsistent and our hypothesis is dependent on whether the ICN donor was neurotized to native muscle or an FFMT.

The objectives of this study were to (1) document a consecutive single-surgeon series using ICN donors, (2) compare native muscle reinnervation procedures with FFMT reconstruction of elbow extension in traumatic brachial plexus palsies, (3) evaluate power outcomes after long-term follow-up using the Medical Research Council (MRC) power scale, (4) assess for potential prognostic factors that can inform future clinical decision-making. 16

## Methods

We present a consecutive single-surgeon series. Inclusion criteria included patients diagnosed with complete brachial plexus palsies as well as extended partial brachial plexus palsies, where intraplexal donors were insufficient to meet reconstructive needs, and all patients scored M0 for elbow extension power preoperatively. All patients had preoperative electromyographic (EMG) studies and a brachial plexus magnetic resonance imaging to assist in reconstruction planning. Late referrals, or patients whose surgery was delayed for other reasons, were more likely to receive FFMT's because of the duration of denervation of potential target native muscles. Rib fractures were a not an absolute contraindication to exploration of ICN's, but all explored ICNs were selectively stimulated once dissected and isolated to confirm their motor power was intact prior to use. Reconstructions requiring interposition nerve grafts were excluded from this series.

Outcome parameters included time from operation and target movement MRC grade. Functional outcomes were based on the original MRC scale and power grading ≥ M4 were considered “good” or “successful” outcomes, i.e., those with muscle movement against gravity and some resistance as determined on clinical examination. Postoperative EMG were not routinely completed due to potential of sampling error providing unreliable results and the fact that such investigation was not anticipated to direct any future care when clinical examination was clear. 17 “Time to initial movement” is defined as the time between the operation and first recording an MRC grade > 0. “Time to best MRC grade first recorded” is defined as the time between the operation and first recording the best MRC grade the patient ever achieved in the follow-up period. Donor and recipient nerve diameters were measured with a ruler under operating microscope magnification. The average total donor or recipient nerve cross-sectional areas (CSAs) were compiled by calculating the sum of the CSAs of each individual donor or recipient nerve by assuming they were circular and using πr ^2^ to calculate the area of a circle.

Data sets were assessed for normality of distribution using D'Agostino and Pearson normality test. Normally distributed data were analyzed with an unpaired *t* -test with Welch's correction to assess differences between two groups and are expressed as mean (standard deviation, range), whereas a Mann–Whitney test was used to analyze non-normally distributed data and are expressed as median (interquartile range, range). *p* -Values < 0.05 were considered statistically significant. Approval to undertake this research was granted by our institutions Human Research Ethics Committee reference number LLR 061/16. Written informed consent was obtained from all participants in this study.

## Results

A total of 48 intercostal spaces were dissected and 62 separate motor nerve components of these spaces were used across 21 reconstructions ( Table 1 ). Most injuries were caused by motor bike or car collisions ( *n* = 12 and 6, respectively), whereas one patient had a water-skiing accident, one patient was crushed by a concrete slab, and the remaining patient received a high-tackle playing semiprofessional rugby.

**Table 1:** Intercostal nerve transfers to native muscles or free-functioning muscle transfers for elbow extension

	ICN transfers to native muscles	ICN transfers to FFMTs	*p-* Value
Number of reconstructions	16	5	–
Follow-up (mo)	52 (21.9, 21–97)	36 (12.6, 21–53)	0.06
Gender	15 M	5 M	–
Age at injury (y)	30 (9.5, 16–51)	33 (8.7, 20–39)	0.48
Time to operation (mo)	7 (4.6, 1–16)	14 (15.6, 4–41)	0.38
Number of ICN branches used per reconstruction	3.0 (1.7, 1–7)	2.8 (0.8, 2–4)	0.73
Total donor nerve cross-sectional area (mm ^2^ )	6.2 (3.1, 2.4–10.5)	7.8 (1.2, 6.4–9.4)	0.23
Total recipient nerve cross-sectional area (mm ^2^ )	7.3 (5.8, 2.4–21.4)	3.5 (0.5, 3.1–4.0)	0.09
Outcomes ≥ MRC grade M4	75% (12 of 16)	100% (5 of 5)	**<0.05**
Outcomes = MRC grade M3	12.5% (2 of 16)	0% (0 of 5)	0.16
Outcomes < MRC grade M3	12.5% (2 of 16)	0% (0 of 5)	**0.16**
Time to initial movement (mo)	12 (4.7, 5–19)	7 (2.5, 5–11)	**<0.05**
Time to best MRC grade first recorded (mo)	26 (14.1, 11–56)	12 (3.3, 7–15)	**<0.01**

Abbreviations: FFMT, free-functioning muscle transfer; ICN, intercostal nerve; M, males; MRC, Medical Research Council.

Note: All data sets in this table are normally distributed and expressed as mean (standard deviation, range).

For native muscle reconstructions of elbow extension, recipient nerve preference was for more proximal triceps nerves that entered long or lateral heads of triceps to reduce regeneration distance and time. All free-functioning muscle transfers utilized the gracilis muscle. A combination of ICNs from the second to seventh intercostal spaces, including their duplicates, if available, were selected ( Table 2 ). Donor nerves came from no more than three intercostal spaces for any individual reconstruction and those who had more than three donor nerves was due to duplicate motor ICNs being present within an intercostal space and both duplicates being used for transfer.

**Table 2:** Intercostal nerve motor donors

Intercostal space	Number of spaces dissected	% duplicate motor ICNs found (%)	Average diameter of motor donors (mm)
3	8	0	1.50
4	16	0	1.69
5	17	35	1.56
6	6	83	1.54
7	1	100	1.38

Abbreviation: ICN, intercostal nerve.

Overall, successful outcomes ≥ M4 were recorded in 17 of 21 reconstructions. ICN transfers to FFMTs had a significantly higher success rate. There were no significant postoperative donor site complications. No patients were lost to follow-up over a minimum follow-up period of 21 months. Subgroup analysis of the native muscle group between those achieving M4 power and those who did not showed statistically significant difference in time to operation ( Table 3 ).

**Table 3:** Intercostal nerve transfers to native muscles for elbow extension reconstruction

	Outcomes ≥ MRC grade M4	Outcomes < MRC grade M4	*p-* Value
Number of reconstructions	12	4	–
Follow-up (mo) a	55 (22.3, 31–97)	44 (21.1, 21–72)	0.39
Gender	10 M	4 M	–
Age at injury (y) a	29 (10.0, 16–51)	31 (9.0, 20–40)	0.79
Time to operation (mo) a	5 (3.1, 1–13)	13 (4.5, 6–16)	**<0.05**
Number of ICN branches used per reconstruction a	3.1 (1.9, 1–7)	2.8 (1.0, 2–4)	0.66
Total donor nerve cross-sectional area (mm ^2^ ) b	6.3 (3.3, 2.4–10.5)	4.4 (1.8, 3.1–5.7)	0.64
Total recipient nerve cross-sectional area (mm ^2^ ) b	7.9 (6.4, 2.4–21.4)	5.1 (2.8, 3.1–7.1)	0.86
Time to initial movement (mo) b	12 (4.0, 5–16)	13 (9.2, 6–19)	0.89
Time to best MRC grade first recorded (mo) a	27 (16.7, 11–56)	23 (6.2, 19–32)	0.51

Abbreviations: ICN, intercostal nerve; M, males; MRC, Medical Research Council.

a Normally distributed data expressed as mean (standard deviation, range).

b Non-normally distributed data expressed as median (interquartile range, range).

## Discussion

Patients with traumatic BPIs commonly need multiple reconstructions. In our practice, elbow extension reconstruction is prioritized after elbow flexion and multiaxial shoulder movement which, in our experience, are best reconstructed with the potent distal spinal accessory nerve and good quality ipsilateral cervical nerve roots. When intraplexal donors are insufficient to reconstruct elbow extension, such as with complete or extensive partial BPIs, the ICNs are an important extraplexal donor option. They are outside the zone of trauma but in favorable proximity to the limb requiring reconstruction. 4 5 6 The results of this cohort study support the use of ICNs as donors for either native muscle or FFMT reinnervation for reconstruction elbow extension with an overall success rate of 17 of 21 reconstructions. A total of 12 of 16 reinnervated native muscle reconstructions and 5 of 5 FFMT reconstructions achieved a successful M4 outcome. These success rates are substantially higher than the 56% published in a similar-sized study utilizing the less functional ≥ M3 grade as a successful outcome. 13

Successful outcomes are known to decrease as the time from injury to reconstruction increases, with less than 6 months being considered the “golden window.” 18 This was evident in the subgroup analysis of the native muscle group between those achieving M4 power and those who did not, with an average time to operation of 5.4 and 12.5 months, respectively. However, FFMT is not limited by this timeframe and an increased time since injury is strong indication for choosing FFMT over native muscle reconstruction. Expectedly, the average time to operation in the FFMT group was longer at 14 months.

The increased patient morbidity associated with harvesting donor nerves from greater than three intercostal spaces has been reported in the literature. 19 However, we observed duplicate ICN motor branches particularly within intercostal space five (6 of 17 spaces) and six (5 of 6 spaces), which allowed for additional donor nerve harvest without the need to dissect further intercostal spaces.

**Fig. 1:**
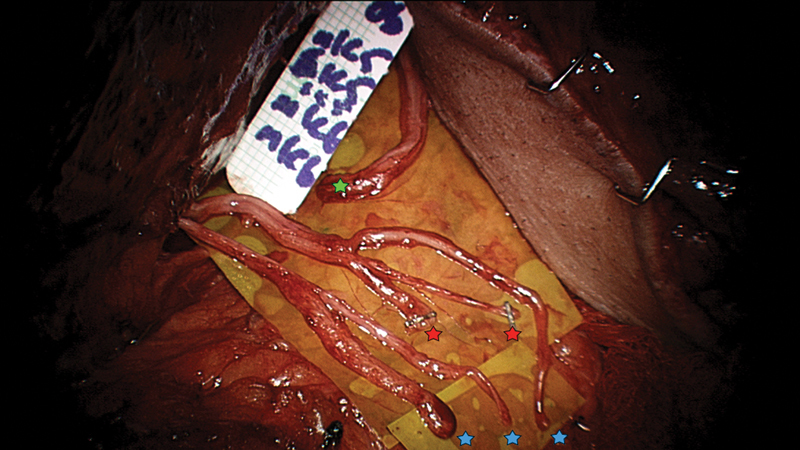
Intraneural neurolysis of intercostal nerves from fifth and sixth spaces to be transferred to gracilis nerve (green) prior to tension-free repairs. Three motor nerves (blue; one from space five and two from space six) to be used, and two sensory nerves (red), unusually both from space five (and none from space six), to be excluded from this motor reconstruction.

CSA of the nerve coaptation is also an important factor in determining the likelihood of successful outcome. 7 While there was no difference in the total donor CSA between the groups, the smaller total recipient CSA in the FFMT did indeed result in a larger donor to recipient ratio in this group. Perhaps more importantly though is the fact that the recipient nerves in the FFMT were all clearly motor nerve fibers heading to the gracilis muscle, whereas a proportion of recipient nerves of the native muscle reconstructions (e.g., radial nerve) may have had sensory fibers inadvertently targeted. This reality has been previously reported as central to the demonstrated greater success of innervating a free gracilis muscle when compared with many recipient targets. 20 We would also like to highlight the importance of intraneural neurolysis of the ICNs to identify and exclude the sensory fascicles from the transfer to ensure more motor nerve fibers are reaching the target muscle ( Fig. 1 ). When sensory fascicles are excluded, ICN motor donor diameters average 1.57 mm (1.00–2.25) depending on the intercostal space dissected ( Table 2 ).

Another fundamental design benefit of FFMT is the ability to position the neuromuscular hilum of the flap close to the donor nerve, thus reducing coaptation to target muscle distance, shortening overall reinnervation time, and eliminating the need for interposition nerve grafting by design. 20 This may be reflected in shorter times to initial movement and best movement in the FFMT group. We describe the time course of muscle recovery using these two parameters as it allows for the easy identification of when recovery has plateaued in each patient.

Adequate follow-up is required to ascertain final outcome. The best power outcome was first recorded at an average of 26 months' postreconstruction in the native muscle group and at 12 months for FFMT group. It is important that patients are informed of the waiting period between surgery and final outcome.

There are a number of limitations of this study. Firstly, there is the possibility that some spontaneous recovery occurred through the parallel native pathways that were deemed injured at the time of operation and left uninterrupted such that regeneration was possible. For example, ICN transfers to triceps nerves and spontaneous recovery to the remaining unoperated nerves to triceps from the radial nerve. Conversely, it is possible that donors or recipients deemed healthy at the time of operation may have actually been involved in the zone of injury. Secondly, we acknowledge that interobserver variability is a risk with clinical assessments, although this was kept to a minimum in this study by all patients being examined and classified to an MRC category by a single surgeon. Thirdly, a larger sample size would increase the power of this study and may enabled further conclusions to be drawn. Finally, for the purpose of comparative statistical analysis, an assumption was made that nerves have a circular CSA, when in practice that is not always the case.

This cohort study showed that timely native muscle reinnervation using ICNs provide M4 power results in the majority of patients; however, ICNs to FFMT was even more reliable and provided M4 power in all patients for reconstructing elbow extension. In the senior surgeon's current practice, if native muscles are uninjured and to be targeted, reconstruction must be as early as possible, with an aim for surgery before 4 months. This is achievable in our major trauma center due to well-organized referral pathways at time of injury and close outpatient follow-up. Surgery later than 4 months is an indication for FFMT. FFMT reconstruction using ICNs is a more complex procedure but has been shown in this study to have faster results and greater reliability in achieving M4 power in our experience. FFMT can generally be performed at any stage after injury in patients with a stable skeleton, supple joints, and adequate soft tissue cover if the patient is cognitively capable and motivated to undertake the required rehabilitation. Due to the experience documented in this paper, the sixth intercostal space is generally dissected first, followed by the fifth or seventh intercostal space depending on the nerve transfer length required in each individual case.
